# *Staphylococcus aureus* Inhibits IL-8 Responses Induced by *Pseudomonas aeruginosa* in Airway Epithelial Cells

**DOI:** 10.1371/journal.pone.0137753

**Published:** 2015-09-11

**Authors:** Samuel M. Chekabab, Richard J. Silverman, Shantelle L. Lafayette, Yishan Luo, Simon Rousseau, Dao Nguyen

**Affiliations:** 1 Research Institute of the McGill University Health Centre, Montreal, Quebec, Canada; 2 Meakins Christie Laboratories, McGill University, Montreal, Quebec, Canada; 3 Department of Medicine, McGill University, Montreal, Quebec, Canada; University of North Dakota, UNITED STATES

## Abstract

*Pseudomonas aeruginosa* (PA) and *Staphylococcus aureus* (SA) are major respiratory pathogens and can concurrently colonize the airways of patients with chronic obstructive diseases, such as cystic fibrosis (CF). Airway epithelial cell signalling is critical to the activation of innate immune responses. In the setting of polymicrobial colonization or infection of the respiratory tract, how epithelial cells integrate different bacterial stimuli remains unknown. Our study examined the inflammatory responses to PA and SA co-stimulations. Immortalised airway epithelial cells (Beas-2B) exposed to bacteria-free filtrates from PA (PAF) induced a robust production of the neutrophil chemoattractant IL-8 while bacteria-free filtrates from SA (SAF) had a minimal effect. Surprisingly, co-stimulation with PAF+SAF demonstrated that SAF strongly inhibited the PAF-driven IL-8 production, showing that SAF has potent anti-inflammatory effects. Similarly SAF decreased IL-8 production induced by the TLR1/TLR2 ligand Pam_3_CysSK_4_ but not the TLR4 ligand LPS nor TLR5 ligand flagellin in Beas-2B cells. Moreover, SAF greatly dampened TLR1/TLR2-mediated activation of the NF-κB pathway, but not the p38 MAPK pathway. We observed this SAF-dependent anti-inflammatory activity in several SA clinical strains, as well as in the CF epithelial cell line CFBE41o-. These findings show a novel direct anti-inflammatory effect of SA on airway epithelial cells, highlighting its potential to modulate inflammatory responses in the setting of polymicrobial infections.

## Introduction

Polymicrobial communities colonize structurally abnormal airways, such as in CF and other chronic obstructive lung diseases, and *Pseudomonas aeruginosa* (PA) and *Staphylococcus aureus* (SA) are the most prevalent pathogens in CF [1]. Concurrent PA and SA infections are found in up to 35% of CF patients [2], as well as in other chronic obstructive lung diseases [3]. Airway epithelial cells (AEC) sense and respond to microbial stimuli through a broad repertoire of patterm recognition receptors (Toll-like and NOD-like receptors) that bind to Pathogen-Asociated Molecular Pattern (PAMPs), non-TLR cell surface receptors (e.g. C-type lectins, TNFR1, EGFR) or through Ca^2+^ dependent signaling or direct cellular damage [4–6]. Activation of the majority of cell-surface TLRs associated with anti-bacterial defenses leads to MyD88 recruitment followed by the activation of four major intracellular signaling pathways: the NF-κB (nuclear factor κ-light-chain-enhancer of activated B cells) pathway and the three MAPK pathways, ERK1/ERK2 (extracellular signal regulated kinases), JNK (c-Jun N-terminal kinases) and p38 MAPK (mitogen activated protein kinase) [7]. While activation of all four of these pathways require the protein kinase TAK1, activation of NF-κB and ERK1/ERK2 is also dependent on activation of the IKK complex [7]. Following TLR-activation, AEC produce pro-inflammatory chemokines and cytokines that recruit and activate innate immune cells essential to the clearance of pathogens [8,9]. Interleukin (IL)-8 (CXCL8), a key neutrophil chemoattractant, is the primary chemokine produced by AEC in response to bacterial stimulation, and IL-8 mediated inflammatory responses are major contributors to the pathogenesis of chronic CF lung diseases. In polymicrobial airway infections, AEC are challenged by a complex array of bacterial signals. How AEC integrate different signals therefore significantly define the immune and inflammatory outcomes during infection.

In this study, we examined the effects of the concurrent stimulation by PA and SA extracellular bacterial products on AEC inflammatory signalling and IL-8 production essential to anti-bacterial defenses. We report that SA filtrates (SAF) significantly inhibited IL-8 production of AEC cultures stimulated by PA filtrates (PAF) or TLR1/2 agonists, and these anti-inflammatory effects were mediated via decreased NF-κB activation. These findings show a novel anti-inflammatory effect of SA on AEC, highlighting its potential to modulate the inflammatory responses by targeting a specific intracellular pathway in the setting of polymicrobial infections.

## Materials and Methods

### Bacterial strains, growth conditions and preparation of filtrates

All bacterial strains used in this study are listed in Table 1. The PA strain PAO1 and SA strain ATCC29213 were used for all experiments unless otherwise specified. To generate PAF and SAF filtrates, bacterial cultures were grown in LB broth (Difco) at 37°C with shaking at 250 r.p.m. for 24h (to OD_600_ ≈ 5.0 for PA and OD_600_ ≈ 6.5 for SA) unless otherwise specified. Where indicated, SA was grown in Tryptic Soy Broth (TSB, Wisent) at 37°C with shaking at 250 r.p.m. Bacterial cultures were centrifuged at 5,000 g for 10 min and the supernatants were sterile filtered with low-protein binding 0.22 μm cellulose acetate filters (Corning) and were stored at -20°C until use. All filtrates were heat inactivated for 10 min at 95°C to minimize AEC toxicity unless otherwise specified. The haemolytic pattern of SA strains was assessed visually after growth on LB or TSB agar plates containing 5% sheep blood.

**Table 1:** Bacterial strains.

Strain	Relevant Characteristics	Reference
*Pseudomonas aeruginosa*		
PAO1	**Wild-type reference strain PAO1**	
PAK	**Wild-type reference strain PAK**	
PA14	**Wild-type reference strain PA14**	
**Δ*fliC***	**flagellin deficient mutant in PAO1 strain**	[10]
*Staphylococcus aureus*		
ATCC29213	**Wild-type *S*. *aureus* strain**	
8325–4	**Wild-type *S*. *aureus* strain**	[11]
*hla*	***hla*::Erm, α-toxin deficient mutant (DU1090) in 8325–4 strain**	[11]
*hlb*	***hlb*::Φ42E, β-toxin deficient mutant (DU5719) in 8325–4 strain**	[12]
1S-1	***S*. *aureus* CF clinical strain**	**This study**
1S-2	***S*. *aureus* CF clinical strain**	**This study**
10S	***S*. *aureus* CF clinical strain**	**This study**
44S	***S*. *aureus* CF clinical strain**	**This study**
134S	***S*. *aureus* CF clinical strain**	**This study**
136S	***S*. *aureus* CF clinical strain**	**This study**
145S	***S*. *aureus* CF clinical strain**	**This study**
203S	***S*. *aureus* CF clinical strain**	**This study**
CF03	***S*. *aureus* CF clinical strain**	**This study**
CF07	***S*. *aureus* CF clinical strain**	**This study**

### Airway epithelial cell culture conditions

Immortalized human bronchial epithelial cells Beas-2B were cultured in Dulbecco’s Modified Eagle Medium (DMEM, Wisent) containing 4.5 g/L D-glucose and supplemented with 10% heat-inactivated fetal bovine serum (FBS, Wisent), penicillin (100 U/mL) and streptomycin (100 μg/mL) at 37°C with 5% CO_2_. Cells were grown to confluence and serum starved overnight to prevent serum-dependent MAPK activation, prior to stimulation with bacterial filtrates and agonists and/or inhibitors. Immortalized CFBE41o- AEC [13] were cultured as submerged monolayers in Eagle’s Minimum Essential Medium (EMEM) containing 1 g/L D-glucose and supplemented with 10% heat-inactivated FBS, penicillin (100 U/mL) and streptomycin (100 μg/mL) at 37°C with 5% CO_2_. All AEC were passaged no more than 30 times in total.

### Airway epithelial cell stimulation

AEC were seeded in 12-well polystyrene tissue culture-treated plates (Corning Costar) at cell densities of 4 x 10^4^ cells/cm^2^. At confluence, AEC were starved in starvation medium (DMEM or EMEM containing 0.5% heat-inactivated FBS media, penicillin streptomycin) for 16 h prior to stimulation. After starvation, PAF and/or SAF were added at the indicated concentrations (2.5 to 10% v/v). AEC were then incubated at 37°C with 5% CO_2_ for 6 h unless otherwise specified. For stimulation with TLR1/2, TLR4 and TLR5 agonists, Pam_3_CysSK_4_, *E*. *coli* LPS, or *S*. *typhimurium* flagellin (Invivogen) were used at the indicated concentrations. For stimulation of the EGFR-dependent pathway, Human Epidermal Growth Factor (hEGF) (Roche Diagnostics) was used at the indicated concentration. AEC were stimulated with LB broth medium as negative control. After stimulation, the conditioned AEC supernatants were collected, centrifuged at 13,000 g for 10 min to pellet cell debris, and stored at -20°C until further analysis.

### IL-8 protein measurements in conditioned AEC supernatants

Extracellular IL-8 protein levels were measured in the conditioned AEC supernatants by ELISA (BD Biosciences) after stimulation with TLR agonists, PAF and/or SAF as indicated.

### Quantitative PCR for gene expression

Beas-2B cells were stimulated with PAF, Pam_3_CysSK_4_ and/or SAF, or LB medium (negative control) for the indicated duration, then washed and resuspended in Trizol (Invitrogen, Carlsbad, CA). Total RNA was extracted and cDNA was synthesized as previously done [14]. To determine IL-8 (CXCL8), CXCL2 and ATF3 mRNA levels, quantitative real-time PCR (qPCR) was performed as previously done [14]. Briefly, the PCR reaction was performed in 96-well plates using SYBR Green-based detection on a Step-One-Plus machine (Applied Biosystems) with the primers listed in S1 Table using the following conditions: 95°C for 10 min, 50 cycles of 95°C for 10 sec, and 60°C for 45 sec. The mRNA levels of GAPDH were used for normalization, and the relative expression ratio (fold induction) of the target gene following AEC stimulation compared to LB medium control conditions was determined from cycle threshold values using the Pfaffl method mathematical model [15].

### NF-κB reporter assay

Beas-2B cells stably transfected with the NF-κB promoter-reporter pGL4.28NF-κB were cultured as described previously [16]. Following stimulation with bacterial filtrates and/or TLR agonists, Beas-2B were lysed, and the supernatant was used in a luciferase assay to determine NF-κB activity as previously done [17]. Briefly, cells were washed twice with ice-cold PBS, incubated with reporter lysis buffer (Promega, Madison, WI) for 5 min then collected by scraping and spun down at 13,000 × g for 3 min. The luciferase assay reagent (20 mM Tricine, 1.07 mM (MgCO3)•4 Mg(OH)2•5H2O, 2.67 mM MgSO4, 0.1 mM ethylene-diamine-tetra-acetic acid, 33 mM dithiothreitol, 270 μM coenzyme A, 0.477 mM D-luciferin, and 0.533 mM adenosine triphosphate) was added to the supernatants in 96-well plates, and the luminescence intensity was measured on a Tecan Infinite M1000 plate reader.

### Immunoblotting and antibodies

Immunoblotting was performed as previously done [16,18]. Briefly, following 45 min stimulation with bacterial filtrates and/or TLR agonists, Beas-2B cells were lysed, and 20 μg of Triton-soluble material was subjected to SDS-PAGE. After transfer to nitrocellulose, the membranes were probed with either anti-phospho-p38 MAPK, anti-phospho-IKKαβ or anti-IκB antibodies that were normalized with anti-total p38 MAPK or anti-GAPDH. Anti-phospho-p38MAPK (Thr180/Tyr182) and anti-total-p38MAPK antibodies (Upstate Biotechnology, NY, USA) were used at 1:1000 dilution. Anti-phospho-IKKα/β (Ser176/180) antibody (Cell Signaling Technology, MA, USA) was used at 1:500 dilution. Anti-phospho-IkBα (Ser32/Ser36) and Anti-GAPDH antibodies (Millipore, MA, USA) were used at 1:1000 and 1:4000 dilutions respectively. Goat anti-rabbit IgG DyLightTM800 (35571) and Goat anti-mouse IgG DyLightTM680 (35518) (Thermo Scientific, IL, USA) were used at 1:15000 dilution. Quantitative analysis of the signals from each antibody was performed using the Li-Cor infrared Odyssey imaging system.

### AEC viability

The viability of AEC monolayers was assessed using the Alamar Blue assay (Invitrogen) according to the manufacturer’s instructions, and the fluorescence intensity was measured at Ex 570nm / Em 585 nm with a fluorescence spectrometer (LS50B, Perkin Elmer). The cytotoxicity of bacterial filtrates on AEC was also measured using the CytoTox 96 Cytoxicity Assay (Promega) according to the manufacturer’s instructions. Briefly, LDH levels released in AEC supernatants were detected colorimetrically at 490 nm using a Bio-Rad Model 680 microplate reader, and the % cytotoxicity was calculated as the relative LDH levels released compared to the maximal LDH levels.

### Statistical analyses

All results, unless otherwise specified, are expressed as mean (±SEM) of independent biological replicates, and comparisons of two groups were performed using an unpaired two-tailed student’s t-test. Comparisons of three or more groups were performed using one-way ANOVA with Bonferroni’s correction. Where indicated, results from several independent experiments were pooled for analysis. A *P* value of ≤0.05 was considered to be statistically significant. Analyses were done with the Prism 6 software (Graphpad, CA).

## Results

### Diffusible bacterial products from PA, but not SA, induce a strong IL-8 response in AEC

**Fig 1:**
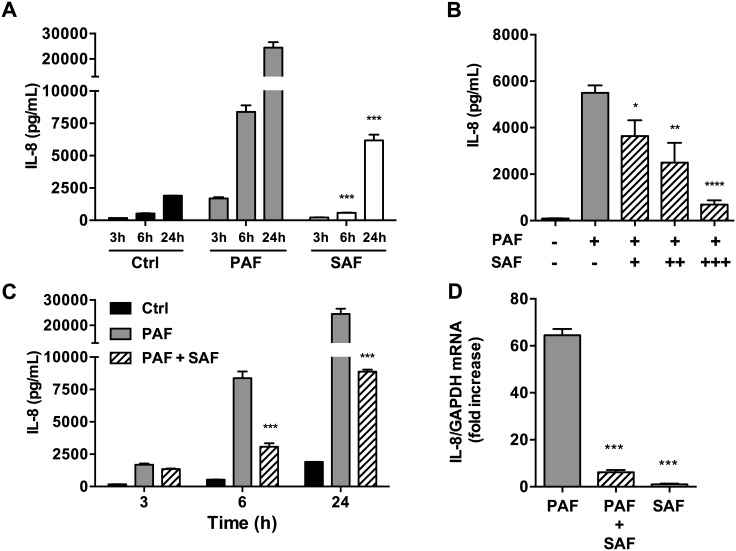
IL-8 response in AECs stimulated with PAF or SAF alone, or PAF+SAF co-stimulation. A) Time-dependent IL-8 response to PAF and SAF. Beas-2B cells were stimulated with PAF (2.5% v/v) and/or SAF (10% v/v) for the indicated duration. B) Dose-dependent IL-8 response to PAF+SAF co-stimulation. Beas-2B cells were stimulated for 6h with PAF (2.5% v/v) and SAF at the following concentration: + = 2.5% v/v; ++ = 5% v/v; +++ = 10% v/v. C) Time-dependent IL-8 response to PAF+SAF co-stimulation. Beas-2B cells were stimulated with PAF (2.5% v/v) and/or SAF (10% v/v) for the indicated duration. D) IL-8 mRNA response to PAF, SAF and PAF+SAF co-stimulation. Beas-2B cells were stimulated with PAF (2.5% v/v) and/or SAF (10% v/v) for 1h. Relative mRNA expression of IL-8 / GAPDH are expressed as fold increase. In A, B and C, extracellular IL-8 levels were measured in the AEC conditioned supernatant by ELISA after stimulation. Stimulation with LB medium was used as control. Results represent the mean (±SEM) of n≥3 independent biological replicates. *P<0.05, **P<0.01, ***P<0.001, ****P<0.0001 compared to PAF alone at the similar time point using 2-way ANOVA (A and C) and 1-way ANOVA (B and D), followed by multiple comparisons Bonferroni correction test.

AEC responds to diffusible bacterial products by activating signalling pathways that leads to pro-inflammatory cytokines production. During colonization or chronic infections, diffusible bacterial products can accumulate in the airways and activate AEC signalling pathways even in the absence of direct cell-cell contact between bacteria and AEC. As done by our group and others, we investigated the cytokine response of Beas-2B monolayers following stimulation with PA filtrates (referred to as PAF) and SA filtrates (referred to as SAF) filtrates which contain diffusible bacterial products [16,19–25]. We focused on the major chemokine IL-8 which is abundantly secreted by AEC and has been extensively implicated in the inflammation of chronic lung diseases such as CF. We measured IL-8 protein levels in the conditions AEC supernatant and observed that PAF induced a robust IL-8 response which increased over time (Fig 1A). On the other hand, the IL-8 response to SAF was considerably weaker compared to PAF (15360 vs. 1380 pg/mL at 6h, P<0.001) (Fig 1A), even at high dose stimulation (10% v/v), suggesting that in our conditions, SAF only elicits a modest IL-8 response.

### SAF inhibits IL-8 protein and mRNA expression during co-stimulation with PAF

Surprisingly, we observed that upon co-stimulation with PAF+SAF, SAF significantly inhibited the IL-8 response to PAF in a dose-dependent manner, with PAF-induced extracellular IL-8 protein levels reduced by more than 90% (P<0.001) at the highest tested dose of SAF (10% v/v) (Fig 1B). In a time course experiment, SAF inhibited PAF-induced IL-8 levels by 71% (P<0.001) at 6h after co-stimulation, and this suppressive effect was sustained at 24h (Fig 1C). In order to examine whether the SAF IL-8 inhibitory effect occurred at the level of mRNA regulation, we measured IL-8 mRNA by qPCR. As expected, IL-8 was highly expressed upon PAF stimulation and minimally expressed during SAF stimulation. Notably, during co-stimulation, SAF nearly abrogated PAF-induced IL-8 mRNA expression, from a ~60-fold to a 6-fold relative expression level (P<0.001) (Fig 1D). These results thus concurred with the secreted IL-8 protein levels measured by ELISA and suggest that SAF inhibits IL-8 at the mRNA expression level. In control experiments, we excluded the possibility that SAF (which contains bacterial proteases) degraded IL-8 by incubating recombinant human IL-8 (rIL-8) with SAF, and detected no cytokine degradation after 24h. Importantly, we also confirmed that SAF and PAF+SAF co-stimulation didn’t caused significant cytotoxicity to AEC using the LDH release and Alamar Blue assay (S1 Fig).

### SAF inhibits the TLR1/2-mediated induction of IL-8

**Fig 2:**
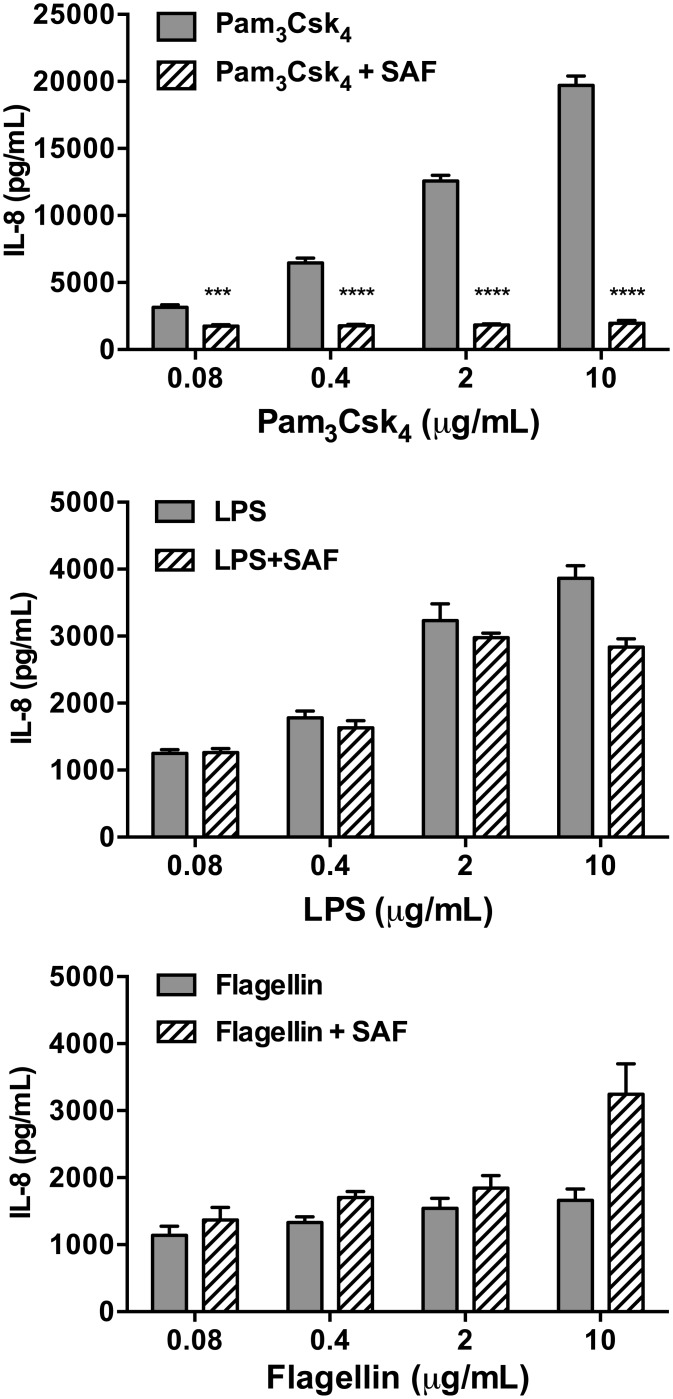
Effects of SAF on TLR agonist-mediated IL-8 responses. Beas-2B cells were stimulated with the TLR agonists Pam_3_CysSK_4_ (TLR 1/2), LPS (TLR4) and flagellin (TLR5) alone or in co-stimulation with SAF (10% v/v) for 6h. Extracellular IL-8 levels were measured in the AEC conditioned supernatant by ELISA after stimulation. Results are shown as mean (±SEM) of four independent biological replicates. ***P<0.001, ****P<0.0001 compared to stimulation with the agonist alone at the same concentration using an unpaired two-tailed student’s t-test.

To determine the pathway through which SAF inhibits PAF-induced IL-8 responses, we first tested the role of the major TLRs expressed by AEC that respond to extracellular bacterial PAMPs, namely TLR1/2, TLR4 and TLR5. We measured secreted IL-8 protein levels in AEC culture supernatants stimulated with Pam_3_CysSK_4_ (a TLR1/2 agonist), LPS (a TLR4 agonist) or purified flagellin (a TLR5 agonist). As shown in Fig 2, Pam_3_CysSK_4_ induced the strongest IL-8 responses, but a dose-dependent IL-8 response was observed with all three TLR agonists. Notably, co-stimulation with SAF strongly inhibited the Pam_3_CysSK_4-_induced IL-8 responses (maximal reduction of 91% with Pam_3_CysSK_4_ 10 μg/mL, P<0.0001) but not LPS nor flagellin-induced IL-8 responses. In addition, SAF did not inhibit IL-8 responses induced by EGFR stimulation, a TLR-independent pathway (S2 Fig), further suggesting that the inhibitory effects of SAF are specific to TLR1/2 (Pam_3_CysSK_4_) mediated IL-8 pathway.

### SAF co-stimulation with Pam_3_CysSK_4_ decreases NF-κB but not p38 MAPK activation by TLR1/2

**Fig 3:**
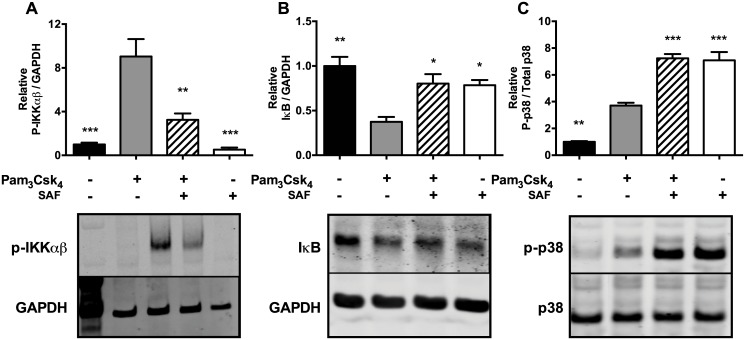
Effects of SAF on IKKαβ, IκB and p38 MAPK. Beas-2B cells were stimulated with Pam_3_CysSK_4_ (10 μg/mL) and/or SAF (10% v/V) for 45 min, and levels of p-IKKαβ (**A**), IκB (**B**) and P-p38MAPK (**C**) were measured in Beas-2B cell by immunoblots. Quantitative analysis of the band signals are shown in the top panels as the mean (±SEM) of three independent biological replicates. Representative blots are shown in the bottom panels. *P ≤ 0.05, **P ≤ 0.01, and ***P ≤ 0.001 compared to the Pam_3_CysSk_4_ alone using 1-way ANOVA, followed by multiple comparisons Bonferroni correction test.

In order to better understand how SAF prevents IL-8 mRNA synthesis, the downstream signalling pathways activated by TLR1/2 were investigated. We measured IKKαβ (subunits alpha and beta of the NF-κB inhibitor kinase IKK) and p38 MAPK phosphorylation, as well as IκB degradation to assess maximal activation of NF-κB and MAPK pathways after 45 min stimulation of Beas-2B cells [26]. As expected, Pam_3_CysSK_4_ stimulation resulted in phosphorylation of IKKαβ (Fig 3A), IκB degradation (Fig 3B) and p38 MAPK phosphorylation (Fig 3C). SAF co-stimulation with Pam_3_CysSK_4_ decreased IKKβ phosphorylation by ~64% (P ≤ 0.01) (Fig 3A) compared to Pam_3_CysSK_4_ alone, and restored IκB levels back to un-stimulated levels (Fig 3B). Interestingly, SAF co-stimulation with Pam_3_CysSK_4_ increased p38 MAPK phosphorylation compared to Pam_3_CysSK_4_ alone (Fig 3C). These results thus suggest that SAF factor(s) that inhibit IL-8 responses act primarily on NF-κB activation without preventing p38 MAPK phosphorylation.

**Fig 4:**
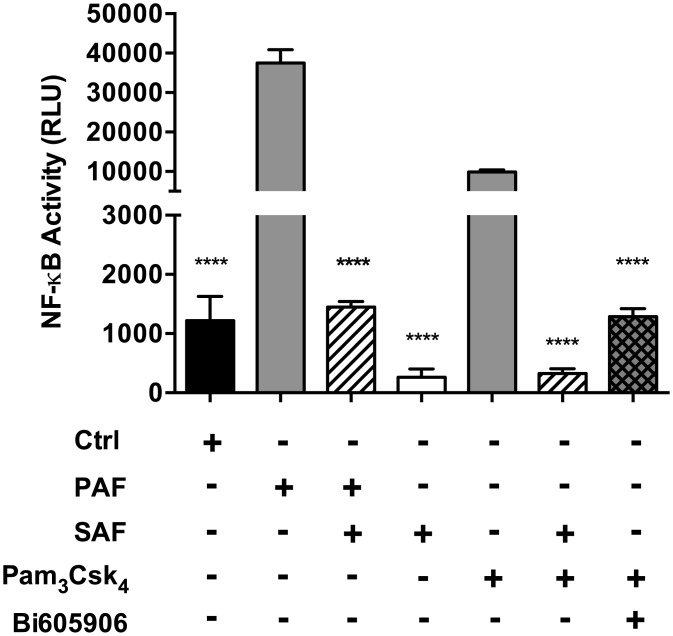
SAF represses PAF and TLR1/2 dependent NF-κB activity. Beas-2B cells stably expressing a NF-κB promoter-reporter (pGL4.28NF-κB) were stimulated with PAF (2.5% v/v), Pam_3_CysSK_4_ (10 μg/ml) and/or with SAF (10% v/V) for 3h. Where indicated, Beas-2B cells were pre-treated with the IKKβ inhibitor Bi605906 (7.5 μg/mL) 1h prior to stimulation with Pam_3_CysSK_4_. Following stimulation, cells were lysed, and the relative luminescence (RLU) was measured in the supernatant using a luciferase assay to determine NF-κB activity. LB media was used as a control. Results are shown as mean (±SEM) of n≥3 independent biological replicates. ****P<0.0001 compared to PAF stimulation alone using 1-way ANOVA, followed by multiple comparisons Bonferroni correction test.

In order to confirm the effects of SAF on NF-κB activation, we also used a NF-κB reporter assay in Beas-2B cells stably expressing luciferase under the transcriptional control of NF-κB (Fig 4). Consistent with the results of IKKαβ phosphorylation and IκB degradation, PAF and Pam_3_CysSK_4_ led to a strong induction of the NF-κB reporter activity, while SAF alone did not (mean RLU 37498 (PAF) and 9899 (Pam_3_CysSK_4_) vs 263 (SAF), P<0.0001). Notably, SAF in co-stimulation with Pam_3_CSK_4_ repressed nearly all NF-κB reporter activity (94% reduction compared to Pam_3_CSK_4_ alone, P<0.0001) and to a slightly greater degree than the IKKβ chemical inhibitor, Bi605906 (87% reduction compared to Pam_3_CSK_4_ alone, P<0.0001). Finally, SAF also significantly repressed PAF-induced NF-κB reporter activity (96% reduction compared to PAF alone, P<0.0001).

**Fig 5:**
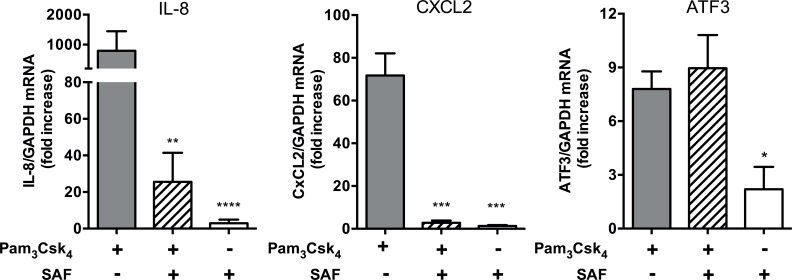
Relative mRNA expression of IL-8, CXCL2 and ATF3 in response to Pam_3_CysSK_4_ alone or in co-stimulation with SAF. Beas-2B cells were stimulated with Pam_3_CysSK_4_ (10 μg/mL) and/or with SAF (10% v/v) for 3h. All results are shown as mean (±SEM) of three independent biological replicates. *P<0.05, **P<0.01, and ***P<0.001 compared to Pam_3_CysSK_4_ alone using an unpaired two-tailed student’s t-test.

Next, we examined the effects of SAF and Pam_3_CysSK_4_ on the NF-κB-dependent and NF-κB-independent gene transcription. IL-8 and CXCL2 (which encodes the CXCL2 cytokine) are both under NF-κB transcriptional control [27,28]. As shown in Fig 5, SAF repressed Pam_3_CysSK_4_-induced IL-8 and CXCL2 mRNA expression by 96% (P<0.01) and 86% (P<0.001) respectively. In contrast, no significant effect was observed on the transcription of ATF3 (activating transcription factor-3 ATF3), which is activated independently of IKKαβ and NF-κB [29]. Taken together, these results suggest that SAF strongly inhibits NF-κB activation and NF-κB-dependent gene transcription, including IL-8.

### The SAF IL-8 inhibitory effects are independent of bacterial surface components, hemolysins but are dependent on bacterial growth medium

**Fig 6:**
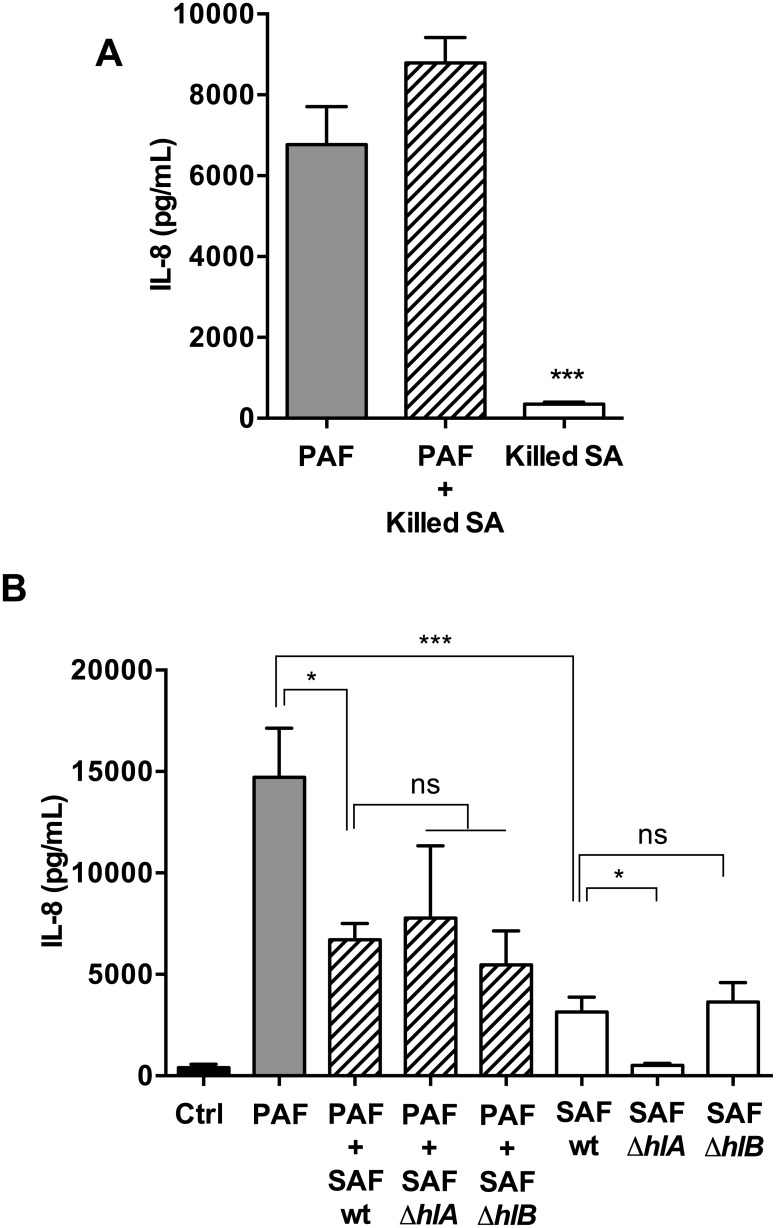
IL-8 response in AEC co-stimulated with PAF and UV killed SA, or SAF from Δ*hla* and Δ*hlb* mutants. **A**) IL-8 response to UV-killed SA. Beas-2B cells were stimulated with PAF (2.5% v/v) and/or a suspension of UV-killed whole SA bacteria (10^8^ CFU/mL) for 6h. **B**) IL-8 response to Δ*hla* and Δ*hlb* SAF. Beas-2B cells were stimulated with PAF (2.5% v/v) and/or SAF (10% v/v) for 6h. PAF and SAF were prepared from bacterial cultures grown in TSB medium for 24h. WT = 8325–4 SA wild-type parental strain; Δ*hla* and Δ*hlb* are its isogenic mutants. In A and B, extracellular IL-8 levels were measured in the AEC supernatant by ELISA after stimulation. Results are shown as mean (±SEM) of four independent biological replicates. *P<0.05, ***P<0.001 compared to PAF alone and to SAF WT, using 1-way ANOVA, followed by multiple comparisons Bonferroni correction test.

SAF contains a complex mixture of secreted and shed bacterial products, and cell wall-associated components such as peptidoglycan-embedded molecules that are detected by TLR2 receptors and induce anti-inflammatory responses [30,31]. In order to determine whether SA cell-wall components were sufficient to inhibit IL-8 responses, we tested AEC stimulated with a suspension of UV killed SA which have intact cell-wall structures. UV-killed SA did not inhibit PAF-induced IL-8 production in co-stimulated AEC (Fig 6A), thus indicating that the SAF anti-inflammatory effects are not attributable to SA cell surface components. Since SA also produces several hemolysins (*hla* and *hlb*) that can modulate the IL-8 induction in AEC and endothelial cells [32–34], we next tested the filtrates of the wild-type SA strain 8325–4, and its isogenic knock-out mutants Δ*hla*, and Δ*hlb* for their ability to repress IL-8 responses. Because SA showed hemolysis during growth in TSB but not LB medium (data not shown), we tested filtrates prepared from SA cultures grown in TSB. In co-stimulation with PAF+SAF, all SA strains inhibited PAF-induced IL-8 responses but there were no significant differences between the wild-type, Δ*hla* and Δ*hlb* SAF (Fig 6B), suggesting that neither Hla nor Hlb are involved. In the experiments with the 8325–4 SA strain, we also noted that TSB-grown SAF alone induced IL-8, in contrast to LB-grown SAF which had minimal IL-8 inducing effects (Fig 1A). In order to determine whether these differences were due to SA strain differences or bacterial growth conditions, we compared filtrates from the wild-type SA (ATCC29213) grown in TSB and LB. We observed that TSB-grown SAF induced IL-8 in a dose-dependent manner, in contrast to LB-grown SAF which had minimal pro-inflammatory effects (S3 Fig), indicating that AEC IL-8 responses to SA were dependent on bacterial factors influenced by growth conditions. We also observed that the SAF anti-inflammatory effects were not attributable to heat-labile bacterial factors (S4 Fig).

### The SAF anti-inflammatory effects are observed with different SA strains and upon co-stimulation with different PA strains

**Fig 7:**
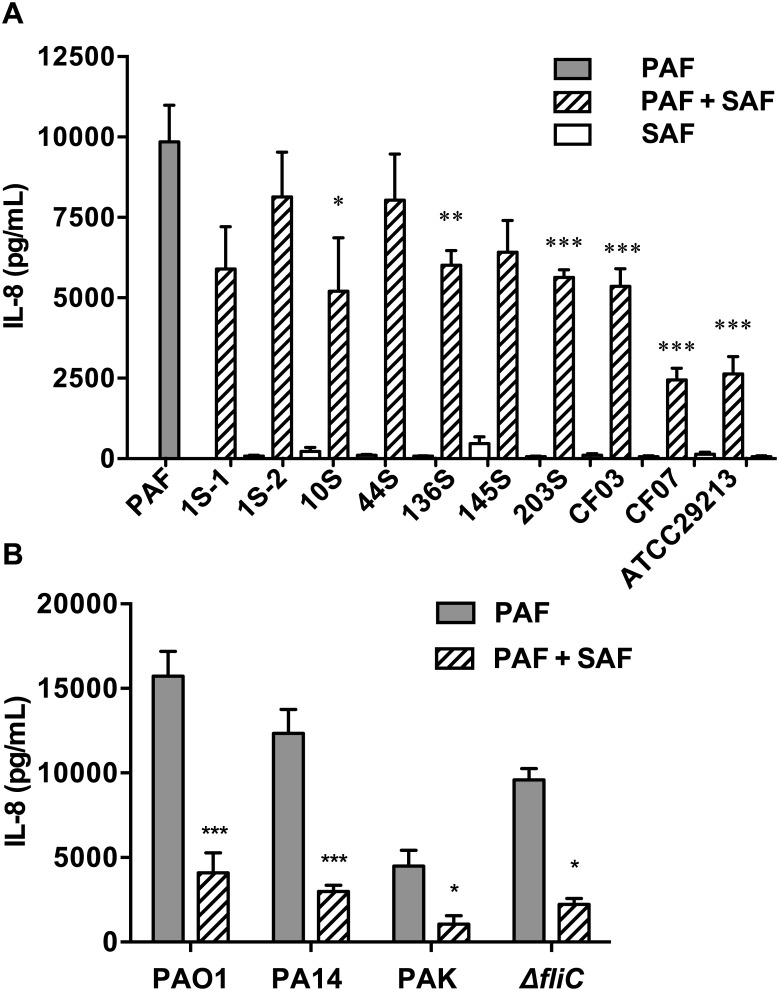
AEC IL-8 responses to co-stimulation with different SA and PA strains. **A**). IL-8 responses to co-stimulation of PAF+SAF from different SA strains. Beas-2B cells were stimulated with SA filtrates (10% v/v) alone or co-stimulated with PAO1 PAF (2.5% v/v) for 6 h. **B**). IL-8 responses to co-stimulation of PAF+SAF from different PA strains. Beas-2B cells were stimulated with filtrates from different PA strains (2.5% v/v) alone, or co-stimulated with ATCC29213 SAF (10% v/v) for 6 h. Extracellular IL-8 levels were measured in the AEC supernatant by ELISA after stimulation. All results are shown as mean (±SEM) of n≥4 independent biological replicates. *P<0.05, **P<0.01, ***P<0.001 compared to PAF alone using an unpaired two-tailed student’s t-test.

In our initial experiments, we tested PAF derived from the commonly used laboratory PA strain PAO1, and SAF from the SA reference strain ATCC29213. In order to validate our findings, we next tested a panel of clinical SA strains isolated from the respiratory sputum of CF patients and generated SAF as previously done with the ATCC29213 strain. As previously observed, stimulation with the filtrates from the different SA clinical strains did not induce any significant IL-8 response on their own (Fig 7A). Upon PAF+SAF co-stimulation of AEC, the different SA clinical strains exhibited varying degree of PAF-induced IL-8 inhibition. While some SA strains (such as 1S-2) had minimal effects on PAF-induced IL-8 response, several others (such as 136S, 145S) reduced PAF-induced IL-8 levels by more than 50%. Next, to validate whether the SAF anti-inflammatory effects were observed in co-stimulation with other PA strains, we tested PAF prepared from several PA reference strains (PAK and PA14) and observed similar results (Fig 7B). Interestingly, the AEC IL-8 response to a PA *ΔfliC* (flagellin-deficient) mutant was still repressed by SAF, further supporting that this anti-inflammatory effects were not mediated through flagellin-dependent pathways.

### Validation in CF AEC

**Fig 8:**
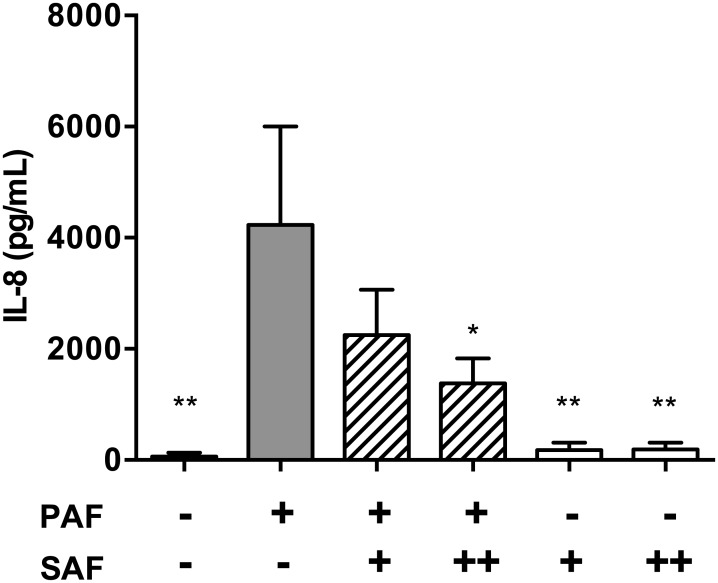
IL-8 responses in CFBE41o- cells stimulated with PAF and SAF. The CFBE41o- airway epithelial cells (CFTR Δ*F508* homozygotes) were stimulated for 6h with PAF (2.5% v/v) and/or SAF at the following volumes. + = 2.5% v/v; ++ = 5% v/v;. Extracellular IL-8 levels were measured in the AEC supernatant by ELISA after stimulation. Results are shown as mean (±SEM) of four independent biological replicates. *P<0.05, **P<0.01 compared to PAF alone using 2-way ANOVA, followed by multiple comparisons Bonferroni correction test.

Concurrent infections with PA and SA are prevalent in the airways of CF patients. Since the IL-8 inflammatory response is a hallmark of CF chronic lung disease, and CFTR mutations in AEC may lead to dysregulated inflammatory signalling, we sought to test the effect of SAF/PAF co-stimulation in a CF-relevant AEC cell lines. We used CFBE41o- cells, which are immortalized bronchial cells homozygous for the ΔF508 CFTR mutation, and measured their IL-8 responses as previously done with Beas-2B cells. As shown in Fig 8, PAF induced IL-8 responses in CFBE41o- cells, while SAF had a moderate inhibitory effect on PAF-induced IL-8 responses, although lower SAF doses were used (2.5% and 5% v/v) to avoid cytotoxicity in CFBE41o- cells.

## Discussion

Polymicrobial communities can colonize the respiratory tract in chronic airway diseases, most notably in CF lung disease. During colonization or chronic infections, bacterial communities typically grow as aggregates within the viscous mucus layer overlying the airway epithelial surface without invasion. As a first line of host defenses, AEC interact with a wide range of microbial products, and their responses are essential for host defense mechanisms and recruitment of innate immune cells. AEC readily detect and respond to shed or secreted bacterial components, and such products stimulate sufficiently the host response without direct bacterial contact [35,36]. AEC stimulation with bacterial filtrates has thus been widely used [16,19–25], and these indirect host-pathogen interactions are modeled in our experimental systems.

AEC responses in polymicrobial infections are poorly understood. This study investigated airways inflammatory responses to complex polymicrobial stimuli. PA and SA, the most prevalent respiratory pathogens, co-localize within the endobronchial lumen [37] and are concurrently isolated from respiratory samples in up to 35% of CF patients [2]. Bacterial products derived from both PA and SA were used for co-stimulation of AEC, and the IL-8 responses were measured. IL-8 is the primary chemokine involved in the neutrophilic inflammation that characterizes CF lung disease, and also the dominant AEC cytokine response to bacteria such as PA [20].

We obtained a robust IL-8 response to PAF that is consistent with previous studies [19,20,38]. During chronic colonization, PA releases many PAMPs, such as lipopeptides, LPS and flagellin which stimulate the AEC IL-8 production through TLR2, TLR4 and TLR5-mediated intracellular signalling pathways respectively [38–41]. Our results showed ~40% less IL-8 response to the Δ*fliC* PAF (flagellin-deficient mutant) compared to wild-type PAF. This confirmed the role of flagellin, but also indicated that other bacterial factors contributed significantly to the AEC IL-8 response. Stimulation of AEC with Pam_3_CysSK_4_ led to the highest IL-8 response compared to LPS or flagellin, highlighting the responsiveness of TLR1/2 in our experimental model.

Studies have reported divergent results on the inflammatory properties of SA infection on AEC. While we noted that SAF (LB-grown) had anti-inflammatory effects on AEC, consistent with reports that AEC are hypo-responsive to gram-positive bacteria [42], these results also stood in contrast to other reports that SAF is highly pro-inflammatory and induces strong IL-8 production in AEC [4,20,23,36,43,44]. It is worth noting that many of these studies used bacterial filtrates from SA grown in TSB rather than LB medium. The experimental conditions may thus explain the differences in results, as highlighted by our results. Although we can not ascertain which laboratory rich media (TSB or LB) best reflect *in vivo* conditions, our results nonetheless provide an experimental system that demonstrates how SA may exert anti-inflammatory effects on AEC, and the important integration of multiple bacterial stimuli in the AEC inflammatory signalling pathways. In order to recreate nutrient conditions closer to the CF lung, we tested the synthetic cystic fibrosis medium [45] but this medium does not sustain adequate SA growth (data not shown). Although the SA anti-inflammatory factor(s) has not been identified, we know that it is extracellular, diffusible and heat resistant, but is not a cell-surface component nor a hemolysin. Importantly, we validated our findings in multiple SA clinical strains as well as another AEC cell line, the ΔF508 CFTR cells CFBE41o-.

Our results highlighted an antagonistic effect between SAF and PAF or TLR1/2 agonists on NF-κB-dependent IL-8 responses. TLR2 and TLR5 are the major AEC TLR receptors activated by PAF [16,18]. Surprisingly, SAF antagonisms only affected TLR1/2-mediated IL-8 synthesis but not TLR4 nor TLR5. As we have shown that SAF targets NF-κB activation, differences in the mechanism of NF-κB activation or its inactivation must exists between TLR2 and the other TLRs like TLR4 and TLR5, although both signal through similar MYD88-dependent mechanisms. Further investigations are required to solve this intriguing question. In contrast to it inhibitory effects on NF-κB, the presence of SAF stimulated p38 MAPK phosphorylation. Although p38 MAPK is required for IL-8 synthesis by TLR1/TLR2 [26], it is not sufficient to stimulate IL-8 in the absence of NF-κB activation as shown in this study. The excessive p38 MAPK activation may be a consequence of NF-κB inhibition, which normally induces important negative regulators of TLR-signalling.

Our study established that extracellular factors produced by SA can inhibit AEC inflammatory response to PAF or to TLR1/2 agonist. Whether this occurs in vivo or with live SA remains to be demonstrated. In our experimental system, prolonged AEC co-cultures with live SA cells led to significant cytotoxicity which precluded meaningful interpretations of the AEC inflammatory signalling and cytokine production in response to bacterial stimuli. Future in vivo studies of PA and SA co-infections would provide valuable insights on the biological significance of our findings.

Many studies have examined PA-SA interspecies interactions, which can be either competitive or cooperative. PA produces several extracellular factors that have antibacterial activities against SA, such as the small molecule HQNO (4-hydroxy-2-heptylquinoline) or LasA protease [46–48]. PA therefore outcompetes SA both *in-vitro* [49], in host cell culture systems [50] and *in vivo* in a mouse model of acute lung infection [51] or wound infection [47]. PA-SA interactions also alter their respective metabolism and gene expression, with consequences on biofilm production [52], antibiotic tolerance [53], and *in vivo* virulence [54,55]. Such interspecies bacterial interactions thus alter bacterial phenotypes, which contribute to the pathogenesis of polymicrobial infections. Our ability to dissect the impact of these bacterial interactions on host responses remains limited, as experimental systems to study polymicrobial infections are only now emerging.

Our studies suggest that the responses of AEC to polymicrobial communities is strongly modulated by the microbial members. For example, polymicrobial infections can cause greater pathology *in-vivo*, such as delayed wound healing in chronic wound infections [56]. We speculate that altered host responses to polymicrobial stimuli may contribute to this. Since SA in CF and chronic obstructive diseases does not typically cause a fulminant pneumonia [37,57,58], we propose that our model may approximate airway colonization or chronic infection, where the presence of SA can modulate AEC responses to other bacterial pathogen such as PA. Since SA is also commonly found in the airway polymicrobial communities, its direct anti-inflammatory effects on AEC may affect the host’s ability to mount an effective protective immune response at early stages of infection, or modulate the neutrophilic inflammatory response during chronic infections.

## Supporting Information

S1 Fig AEC viability following stimulation with PAF and SAF. **A**). Beas-2B cells were stimulated with PAF (2.5% v/v) and/or SAF (10% v/v) for 24h, then treated with 10% Alamar blue for 2h to measure of the cell’s metabolic activity. The relative fluorescence intensity (Ex 570 nm / Em 585 nm) of the AEC supernatant was compared to the negative control condition (LB medium). Results are shown as mean (±SEM) of twelve independent biological replicates pooled from two independent experiments. **B**). Beas-2B cells were stimulated with PAF (2.5% v/v) and/or SAF (10% v/v) for 6h. Cell toxicity was measured using the LDH release assay and results are expressed as % cytotoxicity compared to Trizol-lysed cells (positive controls). LB medium was used as a negative control. Results are shown as mean (±SEM) of twelve independent biological replicates pooled from at least two independent experiments.
(EPS)

S2 Fig Effects of SAF on EGF-dependent IL-8 responses. Beas-2B cells were stimulated with hEGF (0.5 μg/mL) alone, or co-stimulated with SAF (10% v/v) for 6h. LB medium was used as negative control. Extracellular IL-8 levels were measured in the AEC supernatant by ELISA after stimulation. Results are shown as mean (±SEM) of three independent biological replicates.
(EPS)

S3 Fig IL-8 response in AECs stimulated with SAF from SA grown in TSB or LB media. SAF were prepared from bacterial cultures grown in either TSB or LB media for 24h and used at the following volumes: + = 2.5% v/v; ++ = 10% v/v; +++ = 20% v/v. Beas-2B cells were stimulated with SAF for 6h. LB or TSB medium was used as a negative control. Results and are shown as mean (±SEM) of at least two independent biological replicates.
(EPS)

S4 Fig The IL-8 inhibitory effects of SAF are abrogated by heat treatment. Beas-2B cells were stimulated with PAF (2.5% v/v) and/or SAF (10% v/v) for 6h. PAF and SAF were prepared from bacterial cultures grown in LB medium for 24h, and where indicated, SAF was heat treated for 10 min at 95°C. LB medium was used as a negative control. Results are shown as mean (±SEM) of n≥6 independent biological replicates. ***P<0.001 compared to PAF alone using 1-way ANOVA, followed by multiple comparisons Bonferroni correction test.
(EPS)

S1 Table Primers (DOCX)
