# Supplementary material for: Staphylococcus aureus Inhibits IL-8 Responses Induced by Pseudomonas aeruginosa in Airway Epithelial Cells
Source: PLoS One. 2015 Sep 11;10(9):e0137753. doi: 10.1371/journal.pone.0137753 (PMC4567135; doi:10.1371/journal.pone.0137753)
Supplement: S1 Table — (DOCX) [file pone.0137753.s005.docx]

**Table S1. Primers**

| **Primer name** | **Sequence** |
| --- | --- |
| IL8-F | 5’-gtgcagttttgccaaggagt-3’ |
| IL8-R | 5’-ctctgcacccagttttcctt-3’ |
| CXCL2-F | 5’-gcagggaattcacctcaaga3’ |
| CXCL2-R | 5’-gacaagctttctgcccattc-3’ |
| ATF3-F | 5’-aagtgagtgcttctgccatc-3’ |
| ATF3-R | 5’-tttctttctcgtcgcctctttt-3’ |
| GAPDH-F | 5’-agcaatgcctcctgcaccacc-3’ |
| GAPDH-R | 5’-ccggaggggccatccacagtc-3’ |
